# Industrial-Scale Bioconversion of Three-Phase Residue by *Musca domestica* Larvae: Dynamics of Gut Microbiota and Their Ecological Driver

**DOI:** 10.3390/insects16070686

**Published:** 2025-06-30

**Authors:** Wenna Long, Junran Pang, Wantao Yan, Nan Hu

**Affiliations:** College of Biotechnology and Pharmaceutical Engineering, Nanjing Tech University, Nanjing 211816, China

**Keywords:** *Musca domestica*, three-phase residue (TPR), feed–gut–frass continuum, microbial community, lactic acid bacteria (LAB)

## Abstract

The industrial production of *M. domestica* larvae using three-phase residue (TPR) from kitchen waste treatment presents a sustainable circular economy model. Through integrated 16S rRNA sequencing and environmental monitoring in a commercial TPR-based rearing system, we characterized microbiome dynamics across the TPR–gut–frass continuum. The key results showed the following: (1) Larval gut microbiota diversity increased significantly from newly hatched (Md0) to third-instar larvae (Md72), with Shannon and Chao1 indices increasing by 2.01-fold and 6.63-fold, respectively, primarily due to environmental acquisition, although these newly acquired bacteria rarely became dominant. (2) Among the top 10 bacterial genera, 8 were vertically inherited from neonates while 2 originated from TPR, forming a core microbiota comprising *Ignatzschineria*-dominated companion bacteria, *Lactobacillales* members, and functional decomposers (e.g., *Corynebacterium* and *Bacillus*). (3) TPR maintained stable LAB-dominated communities, and distinct successional patterns emerged in both gut and frass microbiota, likely driven by the combined effects of substrate temperature increase, developmental stages, and physicochemical changes. These results enable targeted optimization strategies including LAB-augmented TPR pre-fermentation and functional strain inoculation to enhance bioconversion efficiency.

## Simple Summary

*Musca domestica* larvae have been confirmed as efficient bioconverters for transforming organic waste into high-value protein and fertilizer products. This study systematically characterized the developmental dynamics of gut microbiota in industrially reared *M. domestica* larvae. The results revealed significant restructuring of the larval gut microbiome during development: *Ignatzschineria* dominated as the primary symbiont in the early stage, while lactic acid bacteria (LAB) and functionally specialized microbes involved in nutrient assimilation became predominant in the middle–late stage. Microbial transmission analysis showed that although substantial environmental microbes were acquired during larval growth, the core gut microbiota was primarily established through vertical inheritance from newly hatched larvae, with secondary contributions from feed sources. These findings provide critical scientific guidance for optimizing industrial insect production systems via precision microbiome modulation strategies.

## 1. Introduction

The escalating global population coupled with rising living standards in developing nations has created dual challenges in sustainable food production and organic waste management [1,2]. In this context, saprophagous insects have emerged as valuable bioconversion agents, capable of transforming organic waste back into the food chain [3,4,5,6]. Among these, *H. illucens* and *M. domestica* have shown particular promise as industrial-scale bioconverters, owing to their broad substrate adaptability and rapid life cycles [7,8,9,10]. Their larvae represent nutritionally dense biomass, containing high-quality protein (40–60% dry weight), lipids (15–35%), and chitin (5–15%), along with bioactive compounds including antimicrobial peptides and lectins that enhance their value as animal feed ingredients [11,12]. Furthermore, the residual frass (larval excreta) can be valorized through composting into organic fertilizers, creating an additional revenue stream [13,14]. Recognizing these advantages, numerous countries are now implementing large-scale insect farming systems, with ongoing optimization of production parameters to enhance yield, nutritional quality, and biosafety standards.

The gut microbiota of larvae plays fundamental physiological roles in mediating oviposition behavior, facilitating nutrient digestion, detoxifying xenobiotics, and enhancing immune responses [15,16,17]. Accumulating evidence demonstrates that strategic modulation of gut microbiota through nutritional substrate optimization or probiotic supplementation can significantly enhance microbial community functions. A representative study by Li et al. documented that 10% supplementation with either fruit fermentation broth or lactic acid bacteria (LAB) fermentation broth in kitchen waste substrates selectively promoted probiotic colonization while suppressing ammonifying bacteria populations in *H. illucens* larvae guts, consequently redirecting 22–25% more nitrogen toward larval biomass accumulation rather than ammonia volatilization [18]. Particularly noteworthy are indigenous “companion bacteria” isolated from larval intestines, which upon reintroduction into rearing substrates demonstrate remarkable capacity to remodel gut microbial architecture and improve bioconversion efficiency. The most extensively characterized companion strains for *H. illucens* include *Bacillus subtilis*, *Kocuria marina*, *Lysinibacillus borontolerans*, *Proteus mirabilis*, and *Bacillus velezensis* [19,20,21,22], while *M. domestica* systems benefit from *Enterobacter hormaechei*, *Klebsiella pneumoniae*, *Acinetobacter bereziae*, *Enterobacter cloacae*, *Lysinibacillus fusiformis*, and *Bacillus safensis* [23,24,25]. Additionally, functionally specialized bacteria including *Bacillus subtilis natto*, *Lactobacillus buchneri*, *Arthrobacter* AK19, and *Rhodococcus rhodochrous* 21198 have exhibited comparable probiotic effects in *H. illucens* bioconversion systems [26,27,28]. These collective findings unequivocally establish that the compositional dynamics of larval gut microbiota directly determine critical bioconversion performance metrics, including waste processing rates, nutrient conversion efficiencies, and larval growth yields.

In recent years, China has witnessed growing interest in industrial-scale rearing of *M. domestica* larvae, primarily driven by the abundant availability of three-phase residue (TPR)—a byproduct generated during anaerobic digestion-based treatment of kitchen waste [29,30]. Currently, TPR serves as the predominant feeding substrate for virtually all commercial *M. domestica* farming operations nationwide. Despite this widespread adoption, research addressing TPR bioconversion by *M. domestica* larvae remains limited, particularly regarding characterization of their gut microbiota dynamics. The assembly and succession of larval gut microbial communities are known to be shaped by multiple determinants including substrate nutritional composition [31,32], developmental stage [33], and production scale [26]. Existing studies on *M. domestica* gut microbiota have predominantly employed laboratory-scale systems with wheat bran-based diets [23,24,25,31,34], creating a critical knowledge gap regarding microbial ecology under industrial rearing conditions with TPR substrate. This underscores the necessity for systematic investigation of gut microbiota features in large-scale *M. domestica* production systems utilizing TPR.

This study was conducted under authentic industrial production conditions of *M. domestica* larvae utilizing TPR as the exclusive feeding substrate. Our investigation was designed to address four primary research objectives: (i) characterizing the compositional dynamics and temporal succession of gut microbiota throughout larval development (from newly hatched to third-instar stages), (ii) identifying persistent core microbial taxa that maintain stable colonization, (iii) tracing bacterial transmission pathways along the substrate–gut–frass continuum, and (iv) evaluating the influence of key environmental parameters on microbiota assembly. The findings are anticipated to provide fundamental insights into the gut microbiome ecology of *M. domestica* larvae in commercial-scale operations, while establishing a scientific basis for developing targeted microbiota modulation strategies to enhance bioconversion efficiency.

## 2. Materials and Methods

### 2.1. Industrial-Scale Production of M. domestica Larvae

The industrial-scale production of *M. domestica* larvae was conducted at Heyuetang Modern Agricultural Development Co., Ltd. (Xuyi, China), featuring dedicated infrastructure including four 700 m^2^ rearing workshops, a 300 m^2^ oviposition chamber, and specialized cold storage facilities for product preservation.

**Figure 1:**
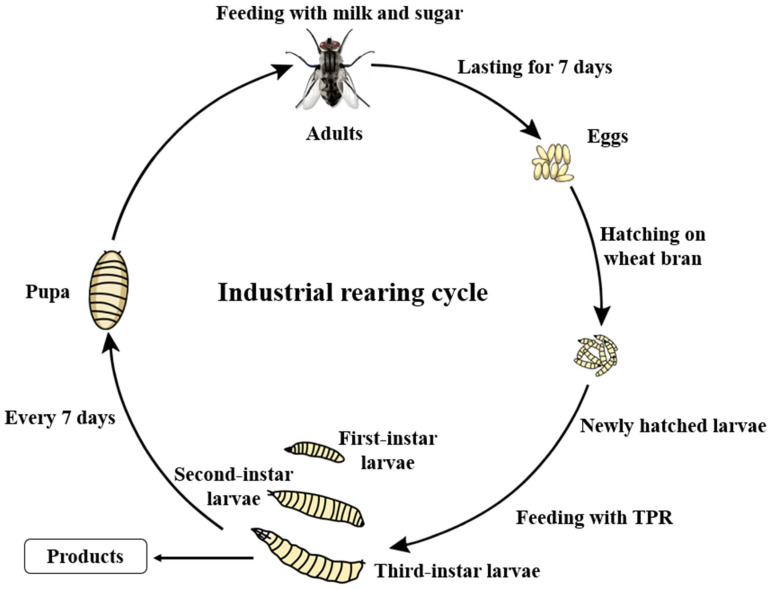
Schematic of *M. domestica* larvae rearing system with TPR-based feeding.

The *M. domestica* production line has maintained continuous industrial operation for over seven years. Adult flies receive a nutritional regimen comprising milk powder, brown sugar, and water. Following egg collection, neonates emerge on moistened wheat bran substrate before being transitioned to TPR-based feeding for a standardized 3-day growth period until harvest. To sustain colony propagation, a subset of third-instar larvae undergoes weekly transfer to the oviposition chamber for maturation into breeding adults. The complete industrial production cycle is schematically presented in Figure 1.

### 2.2. TPR

TPR was sourced from a specialized anaerobic digestion facility processing kitchen waste through environmentally sustainable methods. The treatment process begins with comprehensive pretreatment of kitchen waste involving mechanical sorting, particle size reduction, thermal treatment (boiling), and subsequent three-phase separation [29]. This separation yields three distinct fractions: (1) the lipid fraction, which is recovered for biodiesel synthesis; (2) the liquid slurry fraction, directed to anaerobic digesters for biogas generation; and (3) the solid residual fraction (TPR), which has been increasingly utilized as a nutritionally optimized growth substrate for both *H. illucens* and *M. domestica* larval production systems.

### 2.3. Sampling

The experimental timeline was established with TPR inoculation of newly hatched larvae designated as time zero (0 h). Sampling was conducted at defined intervals: *M. domestica* larvae were collected at 0 h (Md0), 24 h (Md24), 48 h (Md48), and 72 h (Md72); TPR substrates were sampled at 0 h (T0), 24 h (T24), and 48 h (T48); and frass was collected at 24 h (F24), 48 h (F48), and 72 h (F72). Due to biological and operational constraints—specifically, the absence of frass production at 0 h and cessation of TPR supplementation after 48 h—F0 and T72 samples were not obtained. For the sampling methodology, Md0 specimens (≈5 g each) were obtained from three independent hatching boxes, whereas all subsequent samples (≈100 g each) were systematically collected from three standardized positions (front, middle, rear) within the industrial rearing pools to ensure representative biological replicates, yielding a total of 30 samples from three replicates per group.

### 2.4. Processing of Larval Samples

All larval specimens underwent standardized surface sterilization comprising a 5 min 70% ethanol wash followed by three sterile water rinses to eliminate exogenous contaminants [34]. Differential processing protocols were applied based on developmental stage: (i) whole-body homogenization for Md0 and Md24 specimens due to their minute size, and (ii) aseptic intestinal dissection for Md48 and Md72 larvae to obtain gut-specific samples. All processed biological materials were immediately flash-frozen and maintained at −20 °C alongside other sample types until nucleic acid extraction.

### 2.5. DNA Extraction and Analysis

Genomic DNA was isolated from all specimens using the TGuide S96 Magnetic Soil/Stool DNA Kit (Tiangen Biotech Co., Ltd., Beijing, China). Amplification of the bacterial 16S rRNA gene V3-V4 hypervariable region was performed using universal primers 338F (5′-ACTCCTACGGGAGGCAGCA-3′) and 806R (5′-GGACTACHVGGGTWTCTAAT-3′) [35], incorporating degenerate bases for comprehensive microbial coverage. PCR amplicons underwent purification using the Omega DNA Clean-Up Kit (Omega Inc., Norcross, GA, USA) followed by precise quantification via Qsep-400 capillary electrophoresis (BiOptic, Inc., New Taipei City, Taiwan). Final library preparation yielded 2 × 250 bp paired-end reads sequenced on an Illumina NovaSeq 6000 platform (Beijing Biomarker Technologies Co., Ltd., Beijing, China), generating high-resolution microbiota profiles. The raw sequencing data were initially processed using Trimmomatic (v0.33) [36] for quality filtering, followed by primer removal with Cutadapt (v1.8.3) [37]. Subsequent analysis employed the DADA2 denoising algorithm [38] through the R package dada2 for rigorous quality control, paired-end read merging, and chimera removal. The resulting high-quality sequences were further processed using the DADA2 pipeline in QIIME2 (v2020.6) [39] to generate representative ASV sequences. Taxonomic classification was performed against the SILVA 138 reference database [40]. All sequencing data have been deposited in the NCBI Sequence Read Archive (SRA) under accession number PRJNA1276252.

### 2.6. Determination of Physical and Chemical Parameters

Environmental and substrate temperatures were monitored using calibrated mercury thermometers. pH measurements were conducted with a precision pH meter (PHS-3C, Yidian Scientific Instrument Co., Ltd., Shanghai, China) following three-point calibration. Moisture content (MC) was determined gravimetrically by oven-drying at 105 °C until constant mass was achieved. NH_4_^+^ concentrations (measured on a wet weight basis) were quantified using an automated ammonia analyzer (5B-3B, Lianhua Yongxing Technology Development Co., Ltd., Beijing, China) with a detection limit of 0.01 mg/L. Total organic carbon (TOC) content was analyzed according to Chinese agricultural industry standard NY/T525-2021. Nutritional composition analysis included (i) crude protein (CP) determination per GB/T6432-2018 (Kjeldahl method), and (ii) crude fat (CF) quantification following GB/T6433-2006 (Soxhlet extraction). The weight of individual larvae was determined from the mean mass value of 100 randomly selected larvae.

### 2.7. Statistical Analysis

Data organization and graphing were performed using Excel 2016 (Microsoft, Redmond, WA, USA) and Origin 2017 (OriginLab, Northampton, MA, USA). Statistical analyses were carried out with IBM SPSS Statistics 26: α-diversity analysis was performed using Student’s *t*-test and Bonferroni correction to analyze significant differences. The physical and chemical parameters were analyzed for significant differences using one-way ANOVA and Tukey’s test. Principal coordinate analysis (PCoA) using relative abundance of ASV was based on the Bray–Curtis distances. Redundancy analysis (RDA) was implemented in Canoco 5 (Wageningen University & Research, Wageningen, The Netherlands) [41]. SourceTracker analysis, based on Bayesian algorithms, was executed in R 3.1.1 (SourceTracker v1.0.1) to identify microbial contamination sources or origins in target samples [42].

## 3. Results

### 3.1. Diversity of Bacterial Communities

The bacterial communities were characterized by sequencing the 16S rRNA gene amplicons. After performing trimming and quality filtering, 2,356,307 clean reads with >99.95% coverage were generated for 30 samples and at least 55,490 clean reads were generated per sample. The rarefaction curves showed that all samples approached the saturation plateau, indicating that the sequencing results truly reflected the composition of the bacterial communities.

The microbial diversity data for all samples are presented in Table 1. A notable increase in bacterial diversity was observed in intestinal samples of *M. domestica*. From newly hatched larvae (Md0) to third-instar larvae (Md72), the Shannon index, Chao1 index, and ASV numbers of gut bacteria increased 2.01-fold, 6.63-fold, and 7.54-fold, respectively. These findings indicate the larval gut’s adaptive capacity to sustain diverse bacterial colonization while effectively acquiring microbes from dietary and environmental sources.

### 3.2. Taxonomic Composition of Bacterial Communities

**Figure 2:**
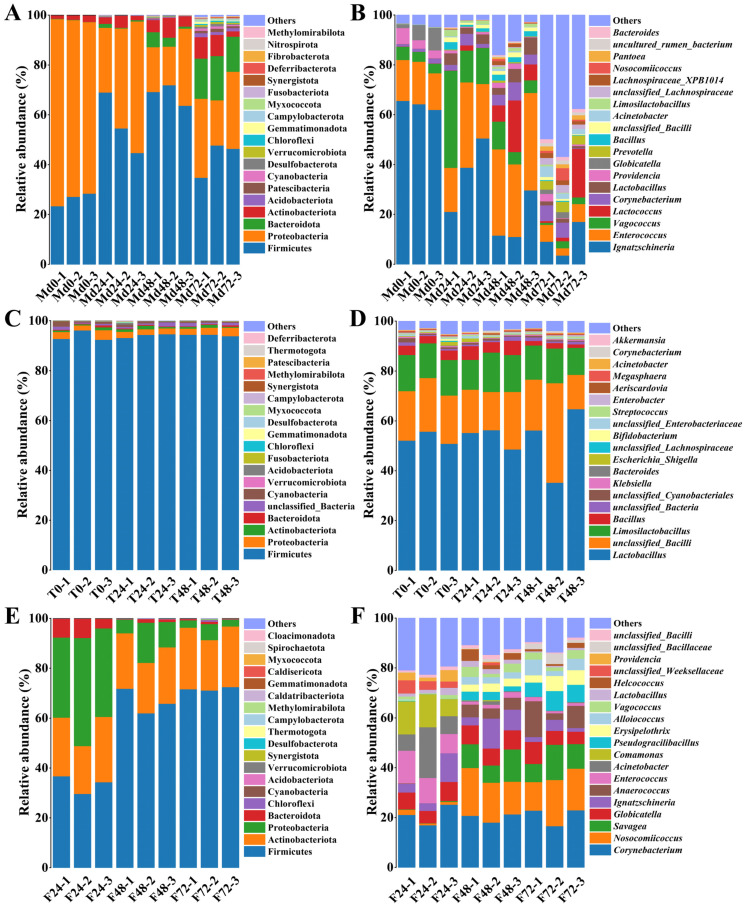
Bacterial relative abundance at phylum and genus levels across larval gut, TPR, and frass samples. (**A**) Phylum-level composition in larval gut microbiota; (**B**) genus-level composition in larval gut microbiota; (**C**) phylum-level composition in TPR microbiota; (**D**) genus-level composition in TPR microbiota; (**E**) phylum-level composition in frass microbiota; (**F**) genus-level composition in frass microbiota.

The gut microbiota of *M. domestica* larvae was predominantly composed of Firmicutes and Proteobacteria at the phylum level. Progressive enrichment of Actinobacteria and Bacteroidetes was observed during larval development (Figure 2A), consistent with previous studies [34,43]. At the genus level, *Ignatzschineria* exhibited remarkable dominance, with its relative abundance reaching 63.95% in newly hatched larvae (Md0) but declining to 9.89% by Md72. Together with *Enterococcus*, *Vagococcus*, *Lactococcus*, and *Corynebacterium*, these taxa formed the core microbiota. Notably, their combined relative abundance decreased from 85.81% (Md0) to 29.03% (Md72), indicating substantial microbial restructuring and increased diversity during maturation (Figure 2B).

The bacterial community in TPR samples was dominated by Firmicutes (>80% relative abundance, Figure 2C), with *Lactobacillus* serving as the most dominant genus (>50% relative abundance across T0-T48). Secondary colonizers include *Limosilactobacillus*, *Bacillus*, and *Bifidobacterium*, which collectively form a stable, LAB-driven microbial consortium (Figure 2D). This compositional profile reflects TPR’s acidic conditions and anaerobic microenvironment, selectively favoring Firmicutes dominance.

The frass microbiota was collectively dominated by the phyla Firmicutes, Proteobacteria, and Actinobacteria (Figure 2E). At the genus level, *Corynebacterium*, *Nosocomiicoccus*, *Savagea*, *Globicatella*, and *Ignatzschineria* constituted the predominant taxa. Their aggregated abundance exhibited an initial marked increase followed by stabilization during frass maturation, demonstrating adaptive microbial succession in response to physicochemical shifts within the frass microenvironment (Figure 2F).

### 3.3. Horizontal Transfer of Bacteria

The sources of larval gut microbiota included vertical inheritance from newly hatched larvae (Md0), acquisition through TPR feeding, and unknown environmental capture. The microbial community in TPR originated from environmental inoculation that occurred during transportation from kitchen waste treatment plants to insect larva farming facilities. Frass primarily inherited its microbiota from the larval gut along with unknown environmental capture. The horizontal transfer of microbes between ecological niches followed two pathways: from TPR to larval gut, and then from larval gut to frass.

**Figure 3:**
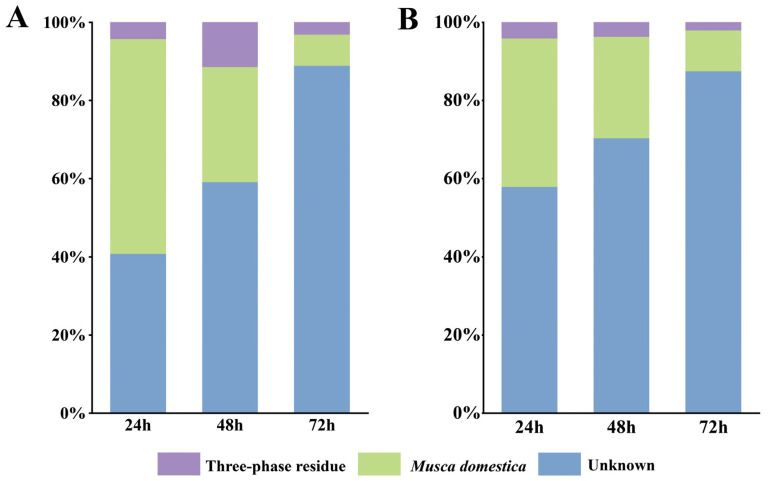
Dynamic source tracking of bacterial communities in larval gut (**A**) and frass (**B**); “*Musca domestica*” indicates larval-native microbiota, “TPR” denotes feed-derived microbiota, and “Unknown” represents environmentally acquired taxa.

SourceTracker analysis of three larval gut samples (Md24, Md48, Md72) and three frass samples (F24, F48, F72) revealed their microbial origins: in Md24, Md48 and Md72, 4.18%, 11.37%, and 3.06% of bacteria were derived from TPR, while 55.03%, 29.46%, and 7.99% came from Md0, with the remainder from unknown environmental capture (Figure 3A). Similarly, in F24, F48, and F72, 4.08%, 3.60%, and 2.01% originated from TPR, while 37.95%, 25.99%, and 10.41% were derived from Md0, with the remainder from unknown environmental capture (Figure 3B). These results demonstrated that microbial horizontal transfer along the TPR–larval gut–frass pathway occurred at relatively low levels, while both the larval gut and frass predominantly inherited their microbiota from newly hatched larvae.

### 3.4. Physicochemical Parameters

The experimental ambient temperature ranged from 27 °C to 33 °C, while substrate temperatures measured 30.8 °C at 0 h, rising progressively to 34.0 °C (24 h), 37.3 °C (48 h), and 39.8 °C (72 h).

The mean individual weights of Md24, Md48, and Md72 were 8.53 mg, 19.89 mg, and 25.78 mg, respectively. As larvae developed, three distinct trends emerged: moisture content (MC) progressively decreased, crude protein (CP) content gradually increased, and crude fiber (CF) content initially rose then declined (Table 2). Due to their minute size, newly hatched larvae (Md0) were excluded from physicochemical measurements.

The physicochemical properties of T0, T24, and T48 remained stable throughout the bioconversion process (Table 2). In contrast, frass samples exhibited significant physicochemical changes: from F24 to F72, the sustained temperature increase led to progressive MC reduction in frass, while NH_4_^+^ production elevated pH values. Comparative analysis revealed lower total organic carbon (TOC) but higher CP concentrations in F48 and F72 relative to F24, likely attributable to combined effects of larval nutritional consumption from TPR and frass microbial metabolism (Table 2).

## 4. Discussion

**Figure 4:**
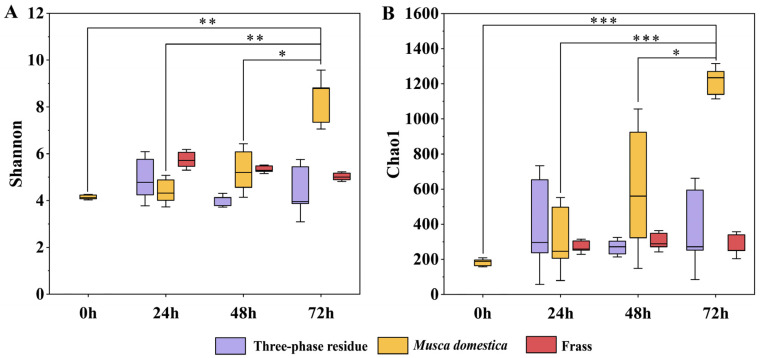
Diversity metrics for three-phase residue, larvae gut, and frass samples. (**A**): Shannon index (diversity); (**B**): Chao1 index (richness). Significance: * *p* < 0.05, ** *p* < 0.01, *** *p* < 0.001.

The α-diversity of bacterial communities was determined using the Chao1 and Shannon indices (Figure 4). During bioconversion, the α-diversity of gut bacterial communities in *M. domestica* larvae increased progressively, while α-diversity changes in frass and TPR remained minimal. Previous laboratory studies demonstrated that gut bacterial diversity in *M. domestica* larvae was consistently lower than observed here [31,34,43], with no significant changes during larval development under controlled conditions [24,25,34]. We propose that industrial rearing maintains a more open microbial environment than laboratory systems, providing richer bacterial inocula that enhance gut microbial diversity throughout larval growth [44]. At present, the products of *M. domestica* larvae are usually used directly as feed for aquaculture, and their high and continuously increasing diversity of gut bacteria poses a risk of pathogen transmission. Therefore, further evaluation of the microbial safety of *M. domestica* larvae products is needed.

**Figure 5:**
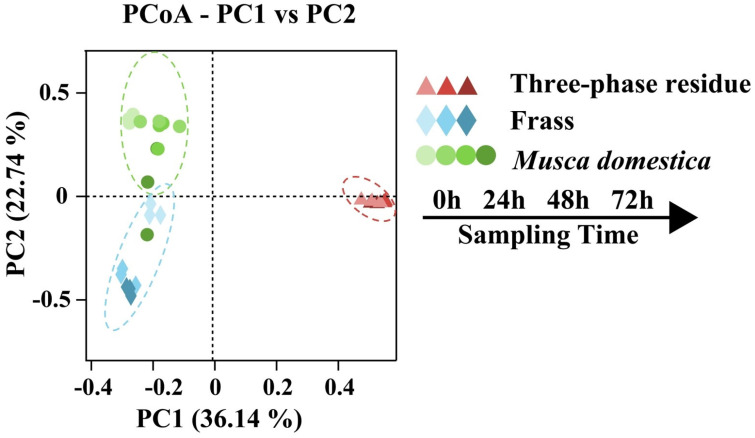
Microbial community similarity analysis. Principal coordinate analysis (PCoA) of operational taxonomic unit (ASV) relative abundance based on Bray–Curtis dissimilarity. Color gradient indicates sampling time points (days), with identically colored symbols representing triplicate samples collected at each time point.

The β-diversity of the bacterial communities between samples was analyzed via PCoA based on the weighted UniFrac distance. As shown in Figure 5, the first two axes accounted for 58.88% of the total variance. Biological triplicate measurements of all samples were highly reproducible. Nine TPR samples were tightly clustered, indicating that their bacterial compositions were stable. The distance between the larval gut samples and the frass samples on the PCoA plots was closer than that of TPR samples, reflecting that they had a more similar bacterial composition.

The SourceTracker analysis revealed that as the larvae developed, both their gut and frass accumulated increasing amounts of environmental bacteria (Figure 3), yet these newly acquired bacteria rarely became dominant species in either niche. At the end of bioconversion, analysis of the top 10 most abundant genera showed that in the larval gut (Md72), *Nosocomiicoccus* and *Lachnospiraceae* came from TPR, while the other eight dominant genera were inherited from newly hatched larvae. A similar pattern was observed in frass (F72), where *Nosocomiicoccus*, *Savagea*, *Anaerococcus*, and *Alloiococcus* originated from TPR, and the remaining six dominant genera derived from newly hatched larvae. These findings indicated that although industrial rearing of *M. domestica* larvae introduced substantial environmental microbes, the initial gut microbiota and the microbial community from feed remained most crucial for maintaining the entire microecosystem.

**Figure 6:**
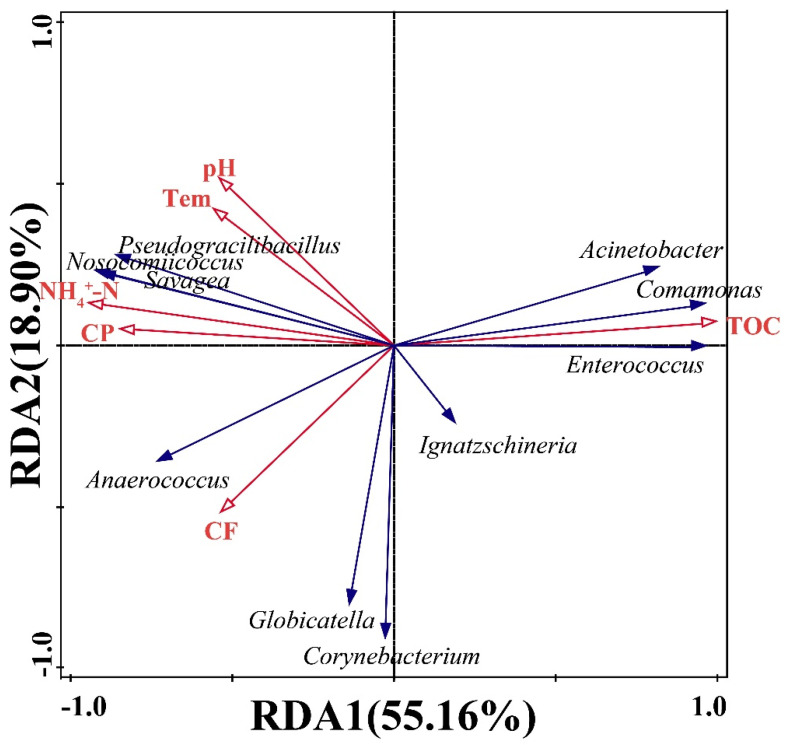
Redundancy analysis (RDA) of top 10 dominant bacterial genera and physicochemical parameters in frass.

A preliminary analysis was conducted on microbial community variations and their influencing factors across the three ecological niches. The microbial composition of TPR remained stable throughout the experimental period (Figure 2D), attributable to its sludge-like viscous properties that restricted aerobic microbial growth, thereby allowing LAB to dominate the TPR microbial structure [45]. Prior to use in this experiment, the TPR had undergone natural inoculation and completed LAB fermentation, resulting in consistently low microbial metabolic activity that maintained stable physicochemical properties (Table 2). The larval gut microbiota underwent significant changes during development, primarily manifested through increasing diversity and shifts in dominant genera (Figure 2B). Given the stable physicochemical properties of daily TPR feeding, we excluded nutritional factors as potential influences and speculated that these changes were driven by substrate temperature elevation (approximately 10 °C increase) and stage-specific microbial selection during larval development [43]. The microbial diversity of frass showed no significant changes, but the relative abundance of dominant genera underwent marked alterations (Figure 2F). The physicochemical properties of frass remained unstable, which correlated with residual nutrient levels from larval digestion of TPR and microbial growth/metabolic activity within frass. RDA of the relationships between physicochemical parameters and dominant microbial genera revealed that *Comamonas*, *Acinetobacter*, and *Enterococcus* exhibited positive correlations with total organic carbon (TOC). In contrast, *Savagea*, *Nosocomiicoccus*, and *Pseudogracilibacillus* were significantly positively correlated with crude protein (CP), ammonium nitrogen (NH_4_^+^-N), temperature, and pH value (Figure 6). Collectively, these results demonstrate that the microbial structure of frass was influenced by variations in physicochemical parameters.

*Ignatzschineria* emerged as the dominant genus within the gut microbiota of *M. domestica* larvae, constituting the highest relative abundance across all bacterial taxa. This predominance was particularly pronounced in newly hatched larvae (Md0), where *Ignatzschineria* reached a relative abundance of 63.95% (Figure 2B). While prior studies have documented the presence of *Ignatzschineria* in *M. domestica* larval guts, none identified it as the most abundant genus [23,34]. Notably, *Ignatzschineria* exhibits broad taxonomic distribution, colonizing the intestinal tracts of diverse insect larvae including *Hermetia illucens* [46,47], *Tenebrio molitor* [48], *Nicrophorus defodiens* [49], and *Bombyx mori* [50]. *Providencia*, another gamma-proteobacterial genus, ranked seventh in relative abundance within the larval gut microbiota. Despite being ubiquitously detected in *M. domestica* larvae across studies, its maximal relative abundance in this study (4.34% in Md0) contrasts with reports identifying *Providencia* as the dominant taxon in other investigations [31,34,51,52]. This discrepancy suggests potential ecological competition between *Ignatzschineria* and *Providencia*, both members of the gamma-proteobacteria class, which may occupy overlapping functional niches during larval development.

LAB constituted a significant functional group within the gut microbiota of *M. domestica* larvae in this study. Among the top 10 dominant genera, five LAB taxa affiliated with the order *Lactobacillales* were identified: *Enterococcus* (second), *Vagococcus* (third), *Lactococcus* (fourth), *Lactobacillus* (sixth), and *Globicatella* (eighth) (Figure 2B). Notably, in the Md48 sample, *Enterococcus* achieved a relative abundance of 34.29%, surpassing even the core genus *Ignatzschineria* (17.40%). The insect larval gut provides an optimal microenvironment for LAB colonization [53,54], though previous studies on *M. domestica* larvae reported lower LAB diversity and abundance compared to this investigation [34,43,45]. We hypothesize that substrate pH critically regulates LAB colonization dynamics. While laboratory studies typically employ neutral-pH wheat bran, the acidified TPR substrate used here appears to selectively favor LAB proliferation, potentially explaining their enhanced establishment in industrial rearing systems.

The genera *Corynebacterium*, *Prevotella*, and *Bacillus* occupied the 5th, 9th, and 10th positions in relative abundance, respectively. From Md0 to Md72, despite an overall increase in bacterial diversity reducing the dominance of genera such as *Ignatzschineria* and *Enterococcus*, *Corynebacterium, Prevotella*, and *Bacillus* exhibited significant increases in relative abundance (Figure 2B). *Corynebacterium* species are reported to exhibit enzymatic capabilities for cellulose and hemicellulose degradation [55,56]; *Prevotella* species possess diverse glycoside hydrolases enabling polysaccharide utilization [57,58]; and *Bacillus* species demonstrate robust enzymatic activities including protease, amylase, and lipase production, which facilitate nutrient breakdown [50]. The colonization success and increased abundance of these taxa in the larval gut may be linked to their potential functional contributions to TPR decomposition and nutrient assimilation in *M. domestica* larvae, based on established metabolic traits in the literature.

Microbial community engineering to enhance insect biomass production represents an emerging frontier in biotechnology [16], with studies demonstrating that strategic enrichment of gut microbiota or exogenous functional bacteria in feed substrates significantly improves larval growth (see Section 1). This study provides the first industrial-scale characterization of gut microbiota composition and succession dynamics in *M. domestica* larvae, establishing critical insights for targeted microbial manipulation. Considering that LAB constituted 50% of dominant gut taxa and TPR’s inherent suitability for LAB fermentation [45], we propose prioritizing LAB strain-specific TPR pre-fermentation to potentially enhance probiotic colonization and growth metrics. Given the documented lignocellulose-degrading capacity of *Actinobacteria* and polysaccharide/proteolytic capabilities of *Prevotella* and *Bacillus* in literature [55,56,57,58,59], controlled substrate inoculation trials could be explored to assess their impact on nutrient assimilation efficiency. Furthermore, mechanistic investigation of *Ignatzschineria*—dominant in Md0 (63.95%)—might be pursued, potentially through CRISPR-based genetic modifications to evaluate symbiotic functionality augmentation. Collectively, these integrated microbial ecology and bioprocess engineering approaches present testable strategies for optimizing industrial insect farming.

## 5. Conclusions

This study presents the first comprehensive analysis of gut microbiome dynamics in *M. domestica* larvae reared at industrial scale using kitchen waste-derived TPR. The study reveals that although significant environmental microbial invasion occurs in the larval gut, the core microbiota is primarily established through vertical inheritance from newly hatched larvae. The dominant symbiont *Ignatzschineria*, whose functional mechanism in larval development remains to be clarified, requires further exploration into leveraging its colonization advantage to promote larval growth. LAB and functional genera involved in nutrient assimilation exhibit significant abundance dominance in the middle–late larval development stages, necessitating pure culture isolation, LAB pre-fermentation, and co-inoculation experiments with functional bacteria to systematically analyze their action mechanisms and evaluate their industrial application potential. Additionally, the increasing gut microbiota diversity may pose risks to product application, calling for urgent biosafety assessments to improve risk management in industrial rearing systems.

## Figures and Tables

**Table 1:** Microbial community richness and diversity estimates from 16S rRNA gene libraries.

Sample	Clean Reads	Number of ASV	Chao1 Estimate	Shannon Index
T0	73,016 ± 316	381 ± 80	395 ± 84	4.92 ± 0.62
T24	72,789 ± 186	255 ± 28	269 ± 29	4.01 ± 0.16
T48	72,457 ± 308	360 ± 53	373 ± 57	4.42 ± 0.72
Md0	72,596 ± 457	159 ± 10	183 ± 13	4.14 ± 0.06
Md24	72,380 ± 296	306 ± 27	316 ± 28	4.41 ± 0.37
Md48	72,245 ± 63	592 ± 42	602 ± 47	5.28 ± 0.62
Md72	73,530 ± 2226	1199 ± 46	1214 ± 54	8.32 ± 0.68
F24	732,623 ± 99	257 ± 22	272 ± 23	5.74 ± 0.24
F48	61,129 ± 1165	286 ± 37	303 ± 33	5.34 ± 0.10
F72	76,563 ± 147	271 ± 39	281 ± 42	5.02 ± 0.11

**Table 2:** Physicochemical characteristics of TPR, larvae, and frass.

Sample	MC (%)	pH	TOC (%)	CP (%)	CF (%)	NH_4_^+^ (g/kg)
T0	76.46 ± 0.12 ^ab^	4.06 ± 0.06 ^a^	37.28 ± 0.52 ^a^	39.23 ± 0.83 ^a^	11.53 ± 0.54 ^a^	0.21 ± 0.01 ^a^
T24	76.14 ± 0.37 ^a^	4.08 ± 0.05 ^a^	36.97 ± 0.71 ^ab^	39.62 ± 0.16 ^a^	11.67 ± 0.33 ^a^	0.23 ± 0.03 ^a^
T48	75.77 ± 0.63 ^a^	4.03 ± 0.06 ^a^	36.09 ± 0.32 ^b^	39.49 ± 0.40 ^a^	11.92 ± 0.79 ^a^	0.26 ± 0.04 ^a^
Md24	79.56 ± 0.02 ^c^	ND	ND	39.85 ± 0.67 ^a^	30.33 ± 2.17 ^b^	ND
Md48	77.46 ± 0.11 ^b^	ND	ND	43.48 ± 0.64 ^b^	32.80 ± 1.99 ^c^	ND
Md72	74.52 ± 0.38 ^d^	ND	ND	48.07 ± 0.35 ^c^	28.95 ± 1.21 ^b^	ND
F24	61.35 ± 0.86 ^e^	6.45 ± 0.38 ^b^	36.46 ± 1.00 ^ab^	30.68 ± 1.31 ^e^	6.69 ± 0.91 ^d^	3.27 ± 0.34 ^b^
F48	60.34 ± 0.42 ^e^	6.54 ± 0.26 ^b^	32.24 ± 0.63 ^c^	35.55 ± 1.24 ^d^	8.35 ± 1.50 ^d^	5.79 ± 0.23 ^c^
F72	58.27 ± 0.52 ^f^	7.21 ± 0.32 ^c^	31.31 ± 0.18 ^c^	34.55 ± 0.52 ^d^	7.40 ± 0.54 ^d^	7.29 ± 0.39 ^d^

MC: moisture content; TOC: total organic carbon; CP: crude protein; CF: crude fat; ND: no detection. The differences were considered statistically significant at different levels (a–f) based on Tukey’s test.

## Data Availability

The original contributions presented in this study are included in the article. Further inquiries can be directed to the corresponding author.
